# Psychological interventions for alcohol use disorders in people living with HIV/AIDS: a systematic review

**DOI:** 10.1186/s13643-019-1176-4

**Published:** 2019-10-28

**Authors:** Munyaradzi Madhombiro, Alfred Musekiwa, James January, Alfred Chingono, Melanie Abas, Soraya Seedat

**Affiliations:** 1 Department of Psychiatry, College of Health Sciences, University of Zimbabwe, Harare, Zimbabwe; 2 Centre for Evidence-based Health Care, Division of Epidemiology and Biostatistics, Faculty of Medicine and Health Sciences, Stellenbosch University, Cape Town, South Africa; 3 Department of Community Medicine, College of Health Sciences, University of Zimbabwe, Harare, Zimbabwe; 4 King’s College London, Centre for Global Mental Health, David Goldberg Centre H1.12, Institute of Psychiatry, Psychology and Neuroscience, King’s College London, De Crespigny Park, London, SE5 8AF UK; 5 Department of Psychiatry, Faculty of Medicine and Health Sciences, University of Stellenbosch, Francie van Zijl Avenue, 7505 Cape Town, South Africa

**Keywords:** Alcohol, HIV, Systematic review, Psychological, Motivational, Cognitive, Screening, Brief, Interventions

## Abstract

**Background:**

Alcohol use disorders (AUDs) in people living with HIV/AIDS (PLWH) are a significant impediment to achieving virological control. HIV non-suppression in PLWH with AUDs is mainly attributable to sub-optimal antiretroviral therapy adherence. Sub-optimal adherence makes control of the epidemic elusive, considering that effective antiretroviral treatment and viral suppression are the two key pillars in reducing new infections. Psychological interventions have been proposed as effective treatments for the management of AUDs in PLWH. Evidence for their effectiveness has been inconsistent, with two reviews (2010 and 2013) concluding a lack of effectiveness. However, a 2017 review that examined multiple HIV prevention and treatment outcomes suggested that behavioural interventions were effective in reducing alcohol use. Since then, several studies have been published necessitating a re-examination of this evidence. This review provides an updated synthesis of the effectiveness of psychological interventions for AUDs in PLWH.

**Methods:**

A search was conducted in the following databases: PubMed, Cochrane Central Register of Trials (CENTRAL), MEDLINE (Ovid), EMBASE, PsychInfo (Ovid) and Clinical trials.gov (clinicaltrials.gov) for eligible studies until August 2018 for psychotherapy and psychosocial interventions for PLWH with AUDs. Two reviewers independently screened titles, abstracts and full texts to select studies that met the inclusion criteria. Two reviewers independently performed data extraction with any differences resolved through discussion. Risk of bias was assessed by two independent reviewers using the Cochrane risk of bias tool, and the concordance between the first and second reviewers was 0.63 and between the first and third reviewers 0.71. Inclusion criteria were randomised controlled trials using psychological interventions in people aged 16 and above, with comparisons being usual care, enhanced usual care, other active treatments or waitlist controls.

**Results:**

A total of 21 studies (6954 participants) were included in this review. Studies had diverse populations including men alone, men and women and men who had sex with men (MSM). Use of motivational interviewing alone or blended with cognitive behavioural therapy (CBT) and technology/computer-assisted platforms were common as individual-level interventions, while a few studies investigated group motivational interviewing or CBT. Alcohol use outcomes were all self-report and included assessment of the quantity and the frequency of alcohol use. Measured secondary outcomes included viral load, CD4 count or other self-reported outcomes. There was a lack of evidence for significant intervention effects in the included studies. Isolated effects of motivational interviewing, cognitive behavioural therapy and group therapy were noted. However for some of the studies that found significant effects, the effect sizes were small and not sustained over time. Owing to the variation in outcome measures employed across studies, no meta-analysis could be carried out.

**Conclusion:**

This systematic review did not reveal large or sustained intervention effects of psychological interventions for either primary alcohol use or secondary HIV-related outcomes. Due to the methodological heterogeneity, we were unable to undertake a meta-analysis. Effectiveness trials of psychological interventions for AUDs in PLWH that include disaggregation of data by level of alcohol consumption, gender and age are needed. There is a need to standardise alcohol use outcome measures across studies and include objective biomarkers that provide a more accurate measure of alcohol consumption and are relatively free from social desirability bias.

**Systematic review registration:**

PROSPERO CRD 42017063856.

## Background

It is estimated that 30–50% of people living with HIV (PLWH) have alcohol use disorders (AUDs) and consequently tend to have unfavourable HIV treatment outcomes [1, 2]. Viral load suppression and testing-and-treating are key targets of the UNAIDS 90-90-90 goals, aimed at eliminating HIV by 2030 [2]. Alcohol use is associated with risky sexual behaviour, sexually transmitted infections and condomless sex, which are all associated with increased transmission of HIV [3–6]. Alcohol use is also associated with reduced uptake of pre-exposure prophylaxis (PrEP) and post-exposure prophylaxis (PEP) [3–6], delayed HIV testing, treatment initiation, reduced adherence to antiretroviral therapy (ART), more treatment interruptions and lack of viral suppression [2]. In addition, alcohol use is associated with traffic accidents, intimate partner violence, liver disease and cancers, which are all associated with premature deaths [7–11].

Evaluation of the extent alcohol use in most settings is usually done through screening with self-report questionnaires and clinical examination. Rarely are laboratory investigations, such as alcohol biomarkers, employed. Alcohol self-report assessments tools include the Cut-Annoyed-Guilty-Eye-opener (CAGE), which is a 4-question screener particularly suited for the presence of dependency and the Alcohol Use Disorders Identification Test (AUDIT), which is a 10-question instrument developed by the World Health Organization (WHO) with scores ranging from 0 to 40 [11–13]. A short form of the AUDIT, the AUDIT-C, is also increasingly used to reduce administration time [14]. Biological measures of alcohol use include blood alcohol concentration (BAC) or surrogates such as liver transaminases, such as gamma glutamyl transaminase (GGT) and mean corpuscular volume (MCV), which are non-specific as they may be changed by liver disease and haematological disease. BAC is, however, able to assess current use but is often unavailable in many settings. Newer biomarkers such as phosphotidyl ethanol (PEth) and ethyl glucuronide (EtG) are promising although the costs may be prohibitive, especially in low-resource settings [15].

Recommended treatments for AUDs include evidence-based therapies such as motivational interviewing, cognitive behavioural therapy, risk reduction, problem-solving techniques, case management and adjunct pharmacological interventions, especially where there is evidence for dependence. Psychological interventions can be delivered in diverse formats, such as individual or group or both. Treatment settings include hospital-based, community, primary care or emergency services [16]. Lately, there has been an increase in the use of smartphones and other mobile devices to deliver these interventions [17], as they increase access in hard-to-reach populations. These technologies are also cost-effective [18, 19].

Given that adherence to ART is the single most important determinant of HIV treatment success, alcohol-focused psychological interventions may significantly improve HIV treatment outcomes [20]. Reviews of interventions that target adherence only, without control of alcohol use, have been inconclusive, leading to calls for interventions that target both adherence and problematic alcohol use [7, 21]. Psychological interventions need to be tailored to address comorbid conditions, such as depression and anxiety, and other psychosocial sequelae (e.g. stigma) that are implicated in poor ART adherence [21, 22]. Psychological interventions may work by addressing the stigma, including self-stigma that PLWH often face. They may also work by assisting PLWH to acquire new problem-solving skills that may be useful in dealing with other life problems. However, the effectiveness of these interventions may be limited by other unresolved psychosocial challenges and the presence of cognitive impairments (e.g. memory impairment) that can lead to unintentional skipping of medication.

Currently, there are insufficient data on the effectiveness of psychological interventions for AUDs in PLWH, specifically with regard to the active ingredients of each intervention, the dosing required, and the circumstances under which they work [16]. It is thus essential that these aspects be teased out in order for firm up treatment recommendations. Brown et al. [16] called for efficacious interventions to be developed and implemented [16]. This systematic review synthesises current evidence on the effectiveness of psychological and behavioural interventions for AUDs in PLWH.

## Objectives

This study aimed to systematically synthesise evidence on the effectiveness of psychological interventions for alcohol use and HIV treatment outcomes in people living with HIV/AIDS with AUDs.

## Methods

The protocol of this review was registered with PROSPERO (CRD42017063856). The review is reported using PRISMA guidelines [23].

### Criteria for considering studies for this review

#### Types of studies

Studies included in the review were randomised controlled trials, including where the control was a waiting list, and designs that used a quasi-random allocation mechanism, such as alternating assignment or next available treatment slot controls.

#### Types of participants

Participants were PLWH aged 16 years and above who had AUDs with or without other substance use and were on ART at hospitals, clinics or in the community.

#### Types of intervention

The interventions included motivational interviewing, motivational enhancement therapy, cognitive behavioural therapy, community contingency therapy, group therapy or any combination of the above that target AUDs, with or without other substance use. Control conditions included adherence counselling, pharmacological detoxification with benzodiazepines, anti-craving medication and referral to psychiatric units or usual care.

#### Types of outcome measures

##### Primary outcomes

Primary outcomes included reduction in alcohol use, as measured by reduction in the frequency and quantity of drinking, binge drinking and heavy episodic drinking; reduction in score on the AUDIT or AUDIT-C or CAGE; blood alcohol concentration average (BAC); peak BAC; and surrogate markers (e.g. GGT and transaminases), in studies that used psychological interventions.

Frequency of alcohol use refers to the number of days alcohol was consumed per a specified period and quantity refers to the amount of alcohol consumed in a specified period.

##### Secondary outcomes

Secondary outcomes were reduction in viral load, CD4 count change, quality of life (as measured by the change in domains including physical health, psychological health, social relationships and environmental health), risky sexual behaviour and ART adherence.

#### Search methods for identification of studies

##### Electronic searches

Two reviewers searched the Cochrane Central Register of Trials (CENTRAL), MEDLINE (Ovid) (1986-; EMBASE (EMBASE.com 1986-), PsychInfo (Ovid) (1986-) and Clinical trials.gov (clinicaltrials.gov/) as at August 2018. There were no language restrictions imposed on the search.

##### Searching other resources

A search of the reference list and bibliographic references of the articles selected for inclusion in the review identified additional relevant articles. These were considered based on their titles and abstracts. Other searches were done through a hand search of authors who have published in psychological interventions for AUDs.

The search terms included thread used in PUBMED, for example, were as follows: (((((HIV[Title/Abstract] OR AIDS[Title/Abstract] OR “human immunodeficiency virus”[Title/Abstract] OR “acquired immunodeficiency syndrome”[Title/Abstract] OR “retroviral infection”[Title/Abstract])) OR (HIV OR “Acquired Immunodeficiency Syndrome”[MeSH Terms]))) AND (((Alcohol*[Title/Abstract] OR drinking[Title/Abstract])) OR (“Alcohol-Induced Disorders” OR “Alcohol-Related Disorders” OR “Alcohol Drinking”[MeSH Terms]))) AND ((“Psychosocial intervention”[Title/Abstract] OR therapy[Title/Abstract] OR psychotherapy[Title/Abstract] OR “motivational interview”[Title/Abstract] OR “motivational interviewing”[Title/Abstract] OR “contingency management”[Title/Abstract] OR “mutual help”[Title/Abstract] OR “twelve step facilitation”[Title/Abstract] OR “twelve steps”[Title/Abstract] OR “twelve step”[Title/Abstract] OR “SBIRT”[Title/Abstract] OR “SBI”[Title/Abstract”))).

### Data collection and analysis

Two reviewers (MM and JJ) independently screened the titles, abstracts, and then full texts to select studies that met our inclusion criteria. The review authors reconciled any differences through discussions and consensus at each stage. MM and JJ who searched the databases and selected the studies achieved a concordance of 0.63, and MM and AM who extracted data on risk of bias assessment achieved a concordance of 0.71.

### Data extraction

Two reviewers (MM and AM) extracted data independently using a pre-piloted data extraction form developed and piloted for this review. Whenever there was any disagreement, the reviewers went through the original articles until they reached consensus. For each included study, we extracted the following: (1) general information (e.g. ethics approval, funding and study period), (2) study design, (3) participants, (4) interventions/comparators, (5) outcomes, (6) results and (7) risk of bias information.

### Assessment of risk of bias in included studies

Two reviewers (MM and AM) independently assessed the risk of bias of the included studies. Differences between the reviewers were resolved through discussion. The Cochrane risk of bias tool was used to assess bias in the included studies [24]. Domains assessed in the risk of bias assessment included selection bias (adequacy of sequence generation and allocation concealment), performance bias (blinding of the participants and research staff) and detection bias (outcome assessors). The other domains assessed were incomplete/missing outcome data caused by attrition or loss to follow-up, publication bias or selective reporting (i.e. where unfavourable or negative outcomes are not reported) and other bias including the influence of funders and other ethical considerations.

### Measure of treatment effect and data synthesis

For binary outcomes, we calculated risk ratios with their corresponding 95% confidence intervals (CI) where raw data were reported, otherwise we reported odds ratios (OR) as reported by the study authors. For continuous data, we calculated mean differences (MD) and corresponding 95% CI. Both RR and MD were calculated using Review Manager 5.3 software. Some studies reported intervention effects for continuous outcomes using Cohen’s *d*, and we reported the effects as such, owing to the fact that there were no sufficient data to report the effects as mean differences. As the outcome measures in individual studies were so diverse, a meta-analysis could not be performed. Analyses were done separately for the different types of interventions.

### Dealing with missing data

In the case of missing data, we used the available case analysis. Where there were missing data, intention-to-treat (ITT) analysis was used.

### Assessment of publication biases

We had planned to plot funnel plots to indicate the possibility of publication bias; however, since no meta-analysis was performed, we did not construct funnel plots.

### Subgroup analysis, investigation of heterogeneity and sensitivity analysis

We had planned to perform a subgroup analysis to identify potential sources of heterogeneity, as well as undertake a sensitivity analysis; however, we did not undertake these as no meta-analysis was performed.

## Results

### Results of the search

**Fig. 1:**
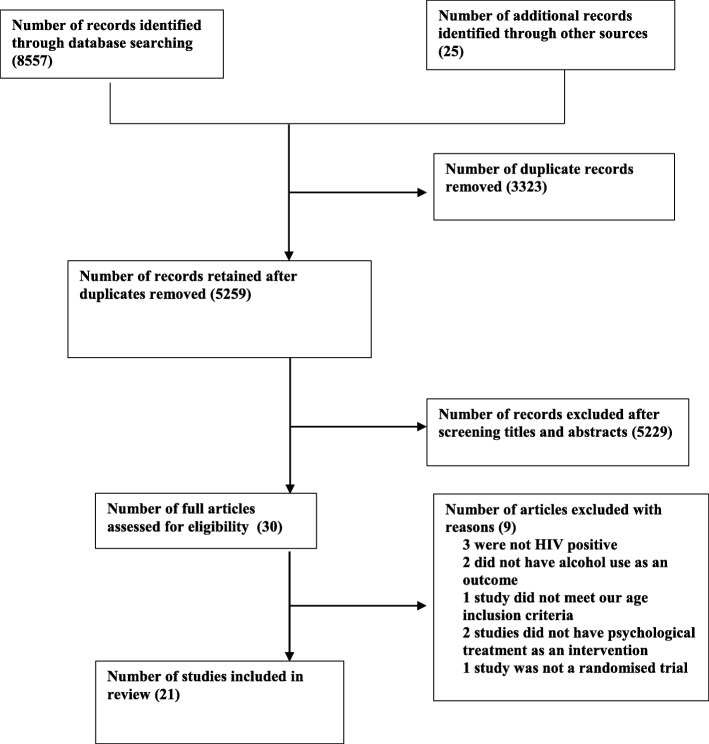
PRISMA study flow

A combined total of 8557 studies were identified through the various search methods, and after removing duplicates, 3258 articles remained. After screening titles and abstracts, full texts of 30 studies were examined and 21 studies with 6954 participants that met the inclusion criteria were included in the review. The PRISMA diagram (Fig. 1) summarises the results of the search.

### Characteristics of included studies

We included 21 studies that assessed alcohol use in PLWH. All 21 studies were randomised controlled trials. Eight studies included both men and women [25–31], three studies included MSM (men who have sex with men) only [32–34] and four studies included women only [35–38]. Seven studies employed various forms of motivational interviewing [19, 26, 29, 30, 32, 33, 39] and three used cognitive behavioural therapy alone or blended with motivational interviewing [27–29]. Four studies evaluated psychological therapies with the addition of technology [19, 25, 26, 40]. All the studies used self-report to measure alcohol use. Table 1 shows a summary of the characteristics of included studies.

**Table 1:** Study characteristics

Study ID	Study design	Participants	Sample size	Intervention	Comparator	Outcome/s
Aharonovich 2017 [19]	RCT	Community sample with SU/AU	47	MI/computer (smartphone)	MI	Number of days drunk and number of drinks per day (TLFB/CIWA-Ar)
Chander 2015 [15]	RCT	HIV-positive women with heavy drinking	148	Brief intervention	TAU	Frequency of drinking
Dawson-Rose 2017 [40]	RCT	18-year-old HIV positive on treatment	208	Computer-administered SBIRT	Physician-administered SBIRT	SSIS (alcohol use)
Gilbert 2008 [25]	RCT	HIV clinic patients	971	Computer/video call	TAU	Frequency of drinking
Hasin 2013 [26]	RCT	HIV-positive patients consuming 3 or more units of alcohol	295	MI + HealthCall	MI	Number of drinks in the last 30 days using TLFB
Kahler 2018 [32]	RCT	MSM	180	MI	TAU	Quantity and Frequency
Meade 2010 [28]	RCT	Young people HIV positive	247	CBT-SM	Support group	Quantity of alcohol
Naar-King 2008 [39]	RCT	16–25-year-old HIV-positive youths	186	Motivational interviewing	Waitlist	Quantity of alcohol use
Papas 2011 [27]	RCT	HIV positive on treatment with hazardous drinking	75	CBT	TAU	% drinking days and mean drinks per day
Parsons 2007 [29]	RCT	HIV positive on treatment hazardous drinkers	143	MI/CBT	Health education condition	Number of drinks per day
Rongkavilit 2013 [41]	RCT	Young PLWH 16–25	110	MI	Health Education	Frequency and quantity of drinking
Rotheram-Borus 2012 [36]	RCT	HIV-positive women	339	Family-based intervention	Waitlist/TAU	Frequency of alcohol use
Samet 2005 [30]	RCT	HIV-positive/with alcohol problems	151	MI	TAU	Alcohol severity and consumption
Samet 2015 [42]	RCT	HIV-positive men with history of drinking and risk sex	700	Group	TAU	Quantity and frequency of drinking
Sikkema 2011 [34]	RCT	MSM	50	Brief intervention and standard care	Standard care	Frequency of alcohol use
Velasquez 2009 [33]	RCT	MSM	253	MI-TTM	Referral to other resources	Quantity, frequency
Wandera 2017 [43]	RCT	PLWH AUDIT-C Score 3 or more	982	Brief Intervention	Positive preventive counselling	AUDIT-C score
Weiss 2011 [37]	RCT	HIV-positive women	482	Individual stress-management SWP II	CBSM/SWPI	Miami Alcohol Use Questionnaire
Wong 2008 [31]	RCT	HIV positive who engaged in risk sex	936	Behavioural intervention	Wait list	Number of days drinking
Yu Liu 2018 [44]	RCT	Newly diagnosed HIV-positive men	367	Peer counselling	TAU	Alcohol consumption and use before sex
Zule 2014 [38]	RCT	Women living with HIV	84	Group therapy	HCT/Nutrition	Number of days abstinent, quantity of drinking, frequency of drinking

Key: *RCT* randomised controlled trial, *SU/AU* substance use/alcohol use, *MI* motivational interviewing, *TAU* treatment as usual, *SBIRT* Screening, Brief Intervention and Referral To Therapy, *PLWH* people living with HIV, *CBT* cognitive behavioural therapy

### Characteristics of excluded studies

There were nine excluded studies. Three studies [45–47] were not conducted in HIV-positive individuals, two studies [34, 48] did not include an alcohol use outcome measure and a single study [49] included young adolescents who did not meet age requirements. Two studies [50, 51] did not include a psychological treatment, and another study [52] was not a randomised controlled trial.

### Risk of bias assessment

**Fig. 2:**
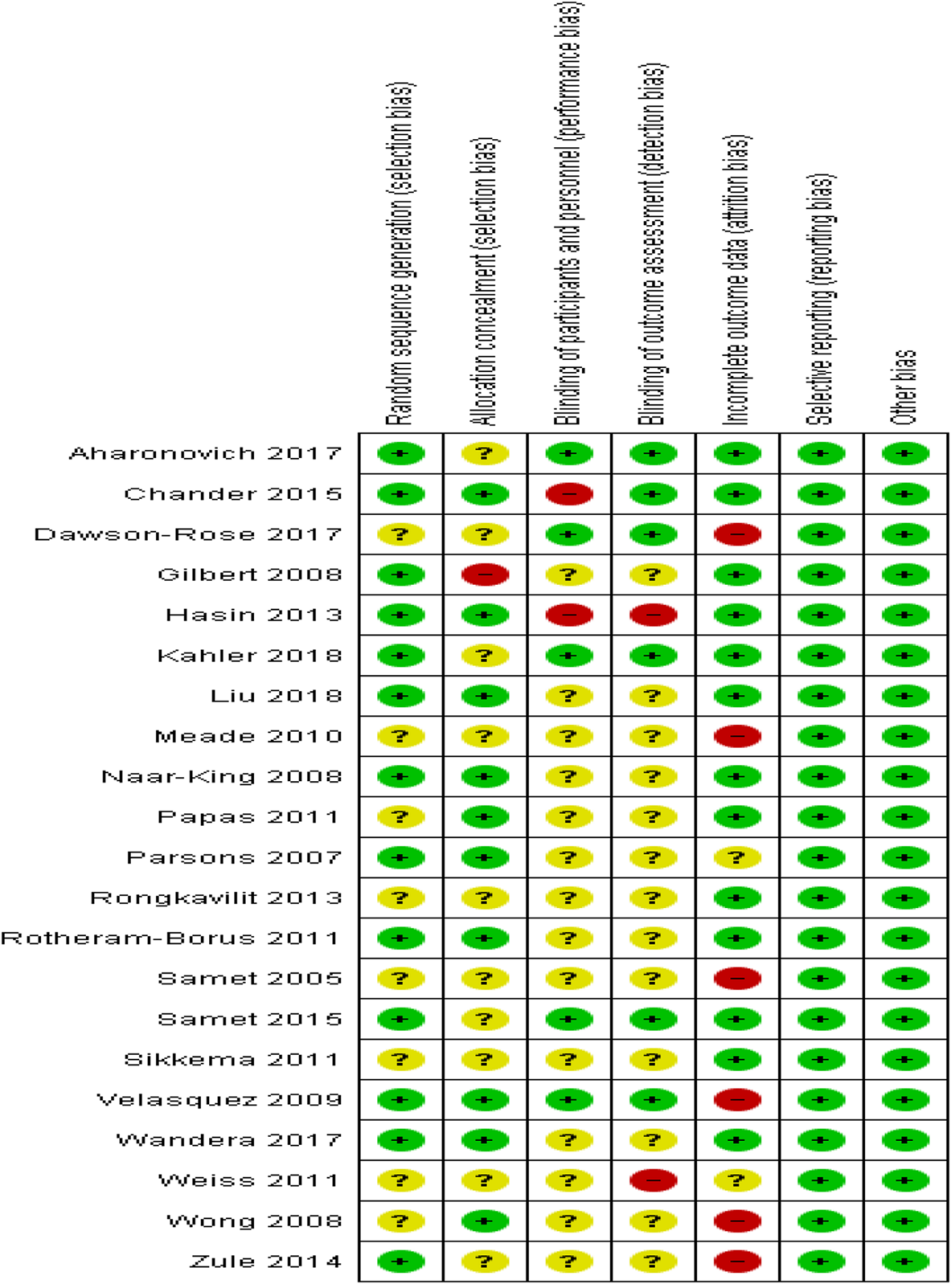
Risk of bias assessment

**Fig. 3:**
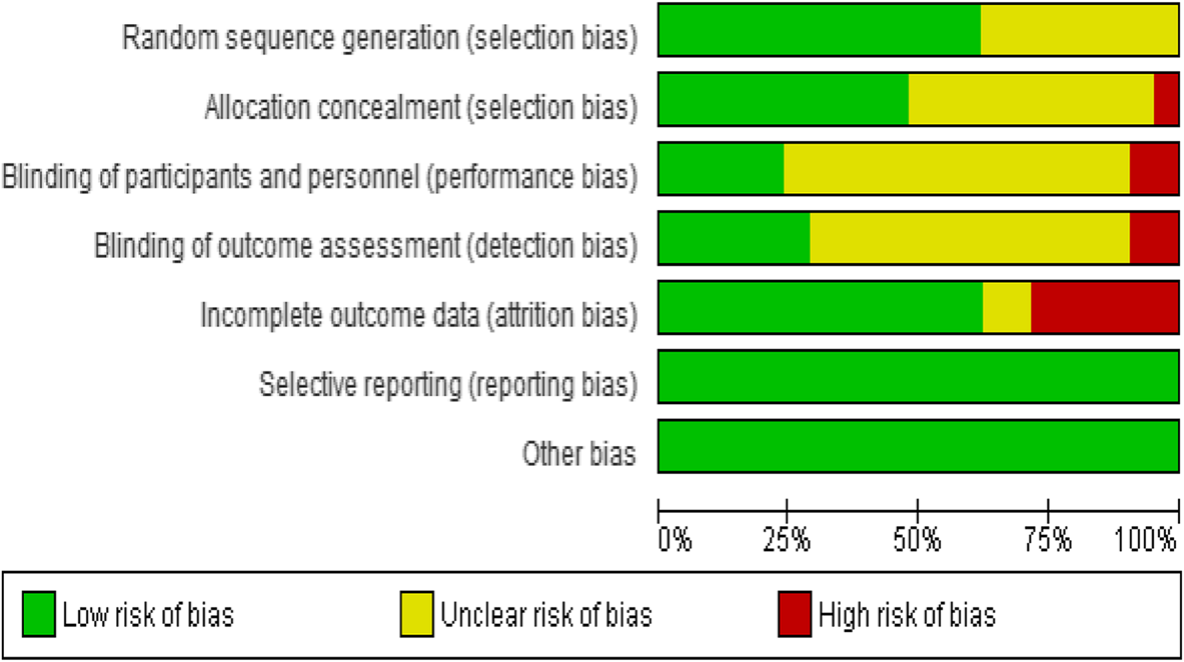
Risk of bias ratios of included studies

The results of the risk of bias assessment are summarised in Figs. 2 and 3. Below we briefly report the results.

#### Sequence generation

Thirteen studies were judged to be at low risk of bias for sequence generation [15, 22, 23, 26, 29, 30, 32, 33, 35, 36, 50–52] because the investigators used computer-generated sequences and run randomisation [53] or SAS in sequence generation, while for eight studies the risk was unclear [24, 25, 27, 28, 31, 34, 37, 53]; for example, Papas et al. [27] stated “randomised” by shuffling “withdrew from the jar a paper with the name of the condition”, while Meade et al. [28] and Samet et al. [30] stated that participants were randomly assigned without explaining how that was done. The remaining studies did not report on sequence generation [25, 27].

#### Allocation concealment

Ten studies were judged as low risk for allocation concealment bias [26, 27, 29, 31, 33, 35, 36, 39, 43, 44] because the investigators stated that they used sealed envelopes to conceal the allocations, 10 had unclear risk [19, 28, 30, 32, 34, 37, 38, 40–42] because they did not report on how they concealed the allocations to the participants and one study was deemed to be high risk [25] because assignment to the intervention group might have been deduced by some patients and their providers through receipt of a computer printout.

#### Blinding (performance bias)

Five studies [19, 32, 33, 40, 42] were judged to be low risk of performance bias because the authors stated that study participants and outcome assessors were blinded and two studies [26, 35] were at high risk of performance bias because they stated that they did not blind the investigators, study participants and outcome assessor. Fourteen studies [25, 27–30, 34, 37–39, 41, 43, 44, 54] were deemed to be of unclear risk of performance bias because they did not report on the blinding of researchers, assessors and participants.

#### Detection bias

Six studies [19, 32, 33, 35, 40, 42] were judged to be at low risk of detection bias because they stated that outcome assessors were blinded. Two studies [26, 37] were judged to be at high risk of detection bias because the assessors were not blinded to the treatment assignments in these studies, Hasin et al. [26] and Weiss et al. [37]. Thirteen studies [25, 27–31, 34, 38, 39, 41, 43, 44, 54] were judged to be of unclear risk because they did not report on the blinding of study outcome assessors or participants carried out self-assessments.

#### Incomplete outcome data (attrition bias)

While 13 studies [19, 25–27, 32, 34, 35, 39, 41–44, 54] were judged to be at low risk of attrition bias because they had low loss to follow-up, it is important to note that losses were proportionate in both arms and investigators used intention-to-treat analysis. Six studies [28, 30, 31, 33, 38, 40] had a high risk of attrition bias (high loss to follow-up), and for two studies [29, 37], it was unclear whether there was attrition bias: Weiss et al. [37] stated that “The overall attrition rate for both studies was approximately 20%” and Parsons et al. [29] indicated that there was a more than 15% loss to follow-up at 6 months in the two treatment arms.

#### Selective reporting (reporting bias)

All 21 studies were judged to be at low risk of selective reporting in this review because all outcomes (as described in “Methods”) were reported in the results.

#### Other potential sources of bias

There was no reason to suspect other biases in the studies included in the review.

### Effects of interventions

The studies assessed the intervention effects using various measures. Table 2 shows the assessed outcomes.

**Table 2:** The outcome data of the included studies

Study ID	Alcohol use measure	Viral load measure	CD4	Adherence	Risky sexual behaviour	Quality of life
Aharonovich 2017 [19]	Frequency (IRR = 0.67, 95% CI = 0.41–1.07). Quantity (IRR = 0.63 (95% CI = 0.36–1.11)	–	–	–	–	–
Chander 2015 [35]	Less likely to have a drinking day (OR 0.42 (95% CI 0.23–0.75) (*p* = 0.005). 90-day drinking frequency in the intervention group was 4.6 [95% CI 0.9, 7.1] Intervention effect 2.9 [95% CI 0.8, 4.4]	Odds ratio 1.30 95% CI 0.65–2.61).	–	(OR 1.11 95% CI (0.853, 1.447) (*p* = .43)). No diff.	Odds of having unprotected vaginal sex compared with the usual care group (AOR = 0.386 with 95% CI (0.156, 0.952), *p* = 0.041)	–
Dawson-Rose 2017 [40]	− 1.59 (95% CI − 2.19, − 1.00)	–	–	–	–	–
Gilbert 2008 [25]	Any drinking at 3 months RR 0.84 (0.651, 1.080) *p* = 0.172 Any drinking at 6 months RR 0.87 (0.666, 1.130) *p* = 0.291 Risk of drinking at 3 month RR 0.88 (0.628, 1.220) *p* = 0.432 Risk of drinking at 6 months 0.85 (0.606, 1.191) *p* = 0.343	–	–	–	Unprotected sex (RR 0.88, 95% CI 0.773, 0.993, *p* = 0.039 at 3 months; and RR 0.80, 95% CI 0.686, 0.941, *p* = 0.007 at 6 months)	–
Hasin 2013 [26]	*χ*^2^, d.f. = 9.11,2, *p* = 0.01)	–	–	–	–	–
Kahler 2018 [32]	Quantity of alcohol use Cohen’s *d* − 5.0 *p* < .001 at 6 months and − 3.3 *p* < 0.04 at 12 months Frequency *d* = −.42 *p* < 01 and .40 *p* < .01 at 12 months	*d* = .02 *p* = .99 at 6 months and *d* = − .11 *p* = .72 at 12 months	*d* = −.25 *p* = .08 at 6 months and *d* = −.21 *p* = .15 at 12 months	–	Condomless sex *d* = −.08 *p* = .79 at 6 months and *d* = −.19 *p* = .10 Sex under influence *d* = −.04 *p* = .20 at 6 months and *d* = −.09 *p* = .11 at 6 months	–
Meade 2010 [28]	Quantity (Wald *χ*^2^(4) = 10.77, *p* < .05)	–	–	–	–	–
Naar-King 2008 [39]	*t*(48) = 1.65, *p* = .05	*t*(45) = 1.91, *p* = .03		–	*t*(47) = .53, *p* = .30	–
Papas 2011 [27]	Percentage daily drinking (*d* = .95, *p* = .0002, mean difference = 24.93 (95% CI 12.43, 37.43) Drinks per drinking day (*d* = .76, *p* = .002, mean difference = 2.88 (95% CI 1.05, 4.70)	–	–	–	–	–
Parsons 2007 [29]	Standard drinks from baseline to 3 months [*F*(1, 112) = 62.7; *p* < 0.001] 6-month follow-up [*F*(1, 93) = 48.7; *p* < 0.001]	(OR = 2.7; *p* = 0.03)	[*F*(1, 115) = 6.44; *p* < 0.02] OR = 3.4; *p* = 0.013)	Percent dose adherence [*F*(1, 107) = 13.5; *p* < 0.001] [*F*(1, 111) = 21.9; *p* < 0.001]	–	–
Rongkavilit 2013 [41]	Frequency *S* = − 0.64, *p* = 0.52 *S* = − 0.84, *p* = 0.40 Quantity *S* = − 0.33, *p* = 0.74 *S* = − 0.79, *p* = 0.43	*t* = 0.75, *p* = 0.47 *t* = − 0.14, *p* = 0.89	–	*S* = − 0.85, *p* = 0.40 *S* = − 0.71, *p* = 0.48	–	Condom use *t* = − 0.87, *p* = 0.39 *t* = − 1.92, *p* = 0.06 Avoiding multiple partners *t* = − 1.00, *p* = 0.32 *t* = − 1.64, *p* = 0.11 HIV disclosure to partners *t* = − 0.18, *p* = 0.86 *t* = − 0.83, *p* = 0.41
Rotheram-Borus 2012 [36]	*t* = − 3.46, df = 256, *p* < 0.01	63% had an undetectable viral load	–	Adherence 76%	–	–
Samet 2005 [30]	No effect on frequency, quantity *p* > 0.25	No effect *p* > 0.25	No effect *p* > 0.25	No effect *p* > 0.25	–	–
Samet 2015 [42]	Quantity OR 1.05 (0.77, 1.43)*p = 0.76* *AOR 1.04 (0.77, 1.40)* *p* = 0.80 Frequency OR 1.00 (0.72, 1.40) *p* = 0.98 AOR 1.00 (0.72, 1.39) *p = 1.00*	Needle sharing OR 1.12 (0.75, 1.69) *p* = 0.58 AOR 1.13 (0.74, 1.73) *p* = 0.56 Distributive needle sharing OR 1.18 (0.75, 1.86) *p* = 0.47 AOR 1.20 (0.76, 1.91) *p* = 0.43	–	–	STI OR 0.65 (0.35, 1.19) *p* = 0.16 AOR 0.63 (0.34, 1.18) *p* = 0.15 Decrease in unprotected sex OR 0.91 (0.69, 1.20 *p* = 0.50 AOR 0.91 (0.69, 1.20) *p* = 0.51	–
Sikkema 2011 [34]	Mean diff. (MD Interv.0.17) (MD Control 0.04 (0.13)	–	–	62.1% at baseline and 57.1% at 6 months	MD 0.16 (− 0.28) 0.44	–
Velasquez 2009 [33]	(Odds ratio [OR] = 1.38; 95% confidence interval [CI] = 1.02–1.86) Higher number of heavy drinking days per 30-day period by a factor of 1.5 (OR = 1.5; 95% CI = 1.08–2.10)	–	–	–	*χ*^2^(33, *N* = 216) = 67.5, *p* < .001 2.19 (OR = 2.19; 95% CI = 1.17–4.11).	–
Wandera 2017 [43]	Mean AUDIT-C difference of the differences = − 0.07, 95% CI − 0.70–0.56, *p* = 0.8266	–	–	–	–	–
Weiss 2011 [37]	Miami Alcohol Use Questionnaire (*F*[2486] = 3.39, *p* < 0.05)	Reduction significant (*p* < 0.01)	–	(*t*[44] = 3.08, *p* < 0.01)		(*p* < 0.05)
Wong 2008 [31]	(*t* = − 15.4, df = 935, *p* < 0.0001) (alcohol and marijuana)	–	–	–	–	–
Liu YU 2018 [44]	23 to 9% (*p* = 0.001)	–	–	–	50 to 16% (*p* < 0.001)	–
Zule 2014 [38]	(Odds ratio [OR] = 3.61; 95% confidence intervals [CI] = 1.23, 11.70; *p* = 0.016)	–	–	–	–	–

### Comparison 1: Motivational interviewing (MI) versus control

Seven studies assessed this comparison [26, 29, 30, 32, 33, 39, 41].

### Primary outcome

#### Alcohol use

##### Quantity of alcohol use

The studies that reported on the quantity of alcohol use did so using different outcomes that did not allow for a meta-analysis. We, therefore, report on the results of individual studies. Kahler et al. [32] found that participants in the motivational interviewing group drank significantly fewer drinks per week compared with the control group at the end of the 6-month treatment period (Cohen’s *d* = − 0.33, *p* < 0.04) [32]. Other studies consistently reported similar effects in the quantity of alcohol use between motivational interviewing and control groups: Velasquez et al. [33] found no significant difference in the average number of drinks per drinking day between motivational interviewing and control groups (OR 1.04, 95% CI 0.77 to 1.40); Naar-King et al. [39] measured most standard drinks in 1 week but found no significant difference in the log-transformed 6-month follow-up change scores between the two treatment groups (MD − 0.36, 95%CI − 0.84 to 0.12, *n* = 49); and Parsons et al. [29] also reported no significant difference in the number of drinks per drinking day at 6 months [29, 33, 39].

##### Frequency of alcohol use

A number of studies measured this outcome; however, the results were reported in different ways that did not allow for a pooling of the data. Kahler et al. [29] found that participants in the motivational interviewing intervention had significantly fewer drinking days per month (Cohen’s *d* = − 0.40, *p* < 0.01), compared to the control group. Hasin et al. [26] found that participants in the motivational interviewing group had a similar number of drinking days as the control group during a 60-day period (MD − 0.71 days, 95%CI − 1.73 to 0.11, *n* = 170), and Rongkavilit et al. [41] also found that participants in the motivational interviewing group had a similar number of drinking days in past 30 days at 6 months as the control group (Mean(SD)[*n*] was 1.0(1.7)[*n* = 49] for intervention and 0.9 (1.5) [*n* = 42] for control) [26, 32, 41].

### Secondary outcomes

#### Reduction in viral load

The results could not be pooled and we report on them individually. All the studies consistently showed similar effects on the reduction in viral load between the intervention and control groups: Kahler et al. [32] found a similar number of participants having detectable viral load (% > 75cp/mL) between the motivational interviewing and control groups at 12 months (RR 0.68, 95% CI 0.20 to 2.33; *n* = 180); Rongkavilit et al. [41] found no significant difference in viral load (log10 copies/ml) at 6 months post session between motivational interviewing and control groups (MD 0.10, 95%CI − 0.53 to 0.73, *n* = 39); Naar-King et al. [39] measured log viral load from baseline to 6 months, and there was no significant difference between motivational interviewing and control groups (MD − 1.23, 95%CI − 2.48 to 0.03, *n* = 46); and Parsons et al. [29] also found no significant differences in log viral load between the two treatment groups at 6 months, according to study authors [29, 32, 39, 41].

#### CD4 count

The two studies reporting this outcome found similar effects on CD4 count between the motivational interviewing and control groups: Kahler et al. [32] found no significant difference in CD4 count between the motivational interviewing and control groups at both 6 months (Cohen’s *d* = − 0.25, *p* = 0.08) and 12 months (Cohen’s *d* = − 0.21, *p* = 0.15), and Parsons et al. [29] also found no significant differences between the two treatment groups at 6 months.

#### ART adherence

The two studies reporting this outcome found similar effects on ART adherence between the motivational interviewing and control groups: Rongkavilit et al. [41] found no significant difference in ART adherence at 6 months (RR 1.02. 95%CI 0.82 to 1.26, *n* = 39) and Parsons et al. [29] measured ART adherence in terms of percent dose adherence but found no significant difference between the two treatment groups at 6 months (MD 5.30%, 95%CI − 7.41 to 18.01%, *n* = 115) [29, 41].

#### Risky sexual behaviour

Naar-King et al. [39] measured the number of unprotected sex acts, but there was no significant difference in the log-transformed 6-month follow-up change scores between the two treatment groups (MD 0.09, 95%CI − 0.54 to 0.72, *n* = 48) [32, 39].

### Comparison 2: Cognitive behavioural therapy (CBT) versus control

One study Papas et al. [27] assessed this comparison [27].

### Primary outcome

#### Alcohol use

##### Frequency of alcohol use

Papas et al. [27] found that participants in the CBT intervention group experienced a significantly greater reduction in the percentage of drinking days in the previous 30 days at the end of 90 days of treatment (MD − 16.92%, 95%CI − 30.46 to − 3.38%, *n* = 68) [27].

### Secondary outcomes

Papas et al. [27] did not measure any secondary outcomes [27].

### Comparison 3: Brief intervention (BI) versus treatment as usual (TAU)

Four studies assessed this comparison [31, 34, 35, 43].

### Primary outcome

#### Alcohol use

##### Quantity of alcohol use

The two studies reporting this outcome found no significant effect for the brief intervention compared to the control group; Chander et al. [35] found no significant difference in the average number of drinks per drinking day between the brief intervention and control groups (RR 0.92, 95%CI 0.68 to 1.24, *p* = 0.586, *n* = 112); Wandera et al. [43] measured alcohol consumption outcomes using change in Alcohol Use Disorders Identification Tool (AUDIT-C) scores but found no significant difference at the end of the treatment period at 6 months (MD 0.50, 95%CI − 0.16 to 1.16, *n* = 320) [35, 43].

#### Frequency of alcohol use

Chander et al. [35] documented a similar 90-day frequency of binge drinking in the brief intervention and control groups (OR 0.60, 95%CI 0.24 to 1.54, *p* = 0.293, *n* = 112) [35].

### Secondary outcomes

#### Reduction in viral load

Chander et al. [35] measured viral suppression (HIV-1 RNA < 50) and found no significant difference between the brief intervention and control groups at 12 months (OR 1.30, 95%CI 0.65 to 2.61, *n* = 148), as reported by study authors [35].

#### ART adherence

Chander et al. [35] measured antiretroviral adherence among the HIV-positive women but found that a brief intervention failed to significantly improve appointment adherence (defined as number of completed visits defined by total scheduled visits) (OR 1.11, 95%CI 0.85 to 1.45, *p* = 0.43, *n* = 148), as reported by study authors [35].

#### Risky sexual behaviour

Chander et al. [35] reported that a brief intervention significantly reduced the likelihood of having unprotected vaginal sex compared to the usual care group (adjusted odds ratio (aOR) 0.39, 95%CI 0.16 to 0.95, *p* = 0.041, *n* = 148), after adjusting for baseline number of days of unprotected sex, as reported by study authors [35].

### Comparison 4: Computer/technology versus treatment as usual (TAU)

Four studies assessed this comparison [19, 25, 26, 40].

### Primary outcome

#### Alcohol use

##### Quantity of alcohol use

Aharonovich et al. [19] compared MI + HealthCall technology versus Attention/Education control and found no significant difference in the number of drinking days during the 60-day period (MD − 1.10, 95%CI − 5.16 to 2.96; *n* = 42) [19].

##### Frequency of alcohol use

Aharonovich et al. [19] found no significant difference in number of standard drinks per day at the end of 60 days of treatment (MD − 0.40, 95% CI − 1.04 to 0.24; *n* = 42) [19].

### Comparison 5: Group versus TAU/wait list/nutritional

Seven studies assessed this comparison [28, 36–38, 42, 44, 54].

### Primary outcome

#### Alcohol use

##### Quantity of alcohol use

Samet et al. [42] measured average drinks per day but found no significant difference between treatment groups (adjusted odds ratio [aOR] 1.04, 95%CI 0.77 to 1.40, *p* = 0.80), according to study authors [42]. Meade et al. [28] measured the reduction in the number of drinks per month from baseline to 12 months and found no significant difference between the ‘coping group’ compared to a support/control group (MD 3.50, 95%CI − 1.98 to 8.98, *n* = 247). Rotheram-Borus et al. [54] assessed mothers living with HIV and their school-going adolescent children using a family-focussed cognitive behavioural group intervention [36]. Among the mothers living with HIV who were using alcohol, those in the intervention unexpectedly drank more than those in the control group (*p* < 0.01) [36].

##### Frequency of alcohol use

Zule et al. [38] found that a greater proportion of participants in the intervention group were abstinent from alcohol compared to the control group (RR 2.57, 95%CI 1.20 to 5.50, *n* = 84) [38].

### Secondary outcomes

#### ART adherence

Weiss et al. [37] SWP II study reported that participants in the intervention group significantly increased ARV adherence compared to the control group (*p* < 0.05) [37].

#### Risky sexual behaviour

Weiss et al. [37] SWP II study reported that the odds of having unprotected sex were significantly reduced in the intervention group compared to the control group (*p* < 0.038) [37]. Samet et al. [42] found no significant difference in the change over time in unprotected sex acts (aOR 0.91, 95%CI 0.69, 1.20), any needle sharing (aOR 1.13, 95%CI 0.74 to 1.73, *p* = 0.51) or STIs (aOR 0.63, 95%CI 0.34 to 1.18, *p* = 0.15) between the two treatment groups [42]. In the Liu et al. [44] study, the intervention reduced the risk of insertive anal sex (aOR 0.65, 95%CI 0.45 to 0.94), condomless anal sex (aOR 0.27, 95%CI 0.10 to 0.74) and illicit drug use (aOR 0.32, 95%CI 0.16 to 0.64), compared to standard of care, at 12-month follow-up [44].

## Discussion

This systematic review aimed to synthesise studies that have investigated the effectiveness of psychological interventions for AUDs in PLWH. We identified 21 studies that met our inclusion criteria. Owing to significant heterogeneity across studies in the populations studied, the interventions tested, and the outcome measures administered, no meta-analysis could be performed. The included studies were randomised controlled trials of PLWH: women only, MSM, mixed gender and adolescents and young adults. Studies aimed at reducing alcohol use in PLWH employed a variety of interventions that included motivational interviewing, CBT, brief interventions, mobile/technology aided treatments and group therapies.

Three previous systematic reviews have reported on the effectiveness of psychological/behavioural interventions on alcohol use in PLWH, published in 2010 [55], 2013 [16] and 2017 [56]. All but one [56] of these concluded that psychological/behavioural interventions were effective for problematic alcohol use in PLWH. New studies have since emerged that address alcohol use in the context of HIV treatment and have been included in this review [2, 26, 38]. In this synthesis, there were no consistent findings of intervention effect for motivational interviewing, compared to a control, on the quantity and frequency of alcohol use, with the exception of the study by Kahler et al. [32]. Papas et al. [27] assessed the effects of cognitive behavioural therapy on the frequency of alcohol consumption and found a significant treatment effect [27]. Neither studies that delivered a brief intervention nor those that administered a technology assisted intervention found significant treatment effects on the quantity and frequency of alcohol use. Of the studies that delivered group therapy, only Zule et al. [38] found intervention effects on alcohol use. Another study documented an increase in the quantity of alcohol consumed in the intervention group [38]. Similarly, secondary outcomes were also heterogeneous and measured in a non-uniform manner across studies, and we were not able to pool data to examine intervention effects on these outcomes.

Across the studies, populations included were diverse. Treatment response may be a function of gender, age and ART adherence. Alcohol users compared to multiple substance users may also respond differently. Some of the interventions were delivered in the community and yet others were delivered at clinics or were hospital based. The context may also affect response to an intervention. Although PROJECT Match was a large study that found that outcomes did not differ by intervention type (motivational enhancement therapy, cognitive behavioural therapy and Twelve-Step Facilitation), the findings of PROJECT Match may not be applicable to diverse HIV-infected populations [57, 58]. Aside from the different theoretical foundations of the aforementioned interventions, differences in treatment duration, number of sessions and delivery agents may contribute to the differences in outcomes recorded in the studies included in this review.

In addition, different measures of alcohol use were employed. Some studies elected to assess the quantity of alcohol consumed within a certain timeframe while other studies assessed the frequency of alcohol consumption. All studies used self-report screeners that are limited by social desirability bias. Few of the studies assessed viral load and CD4 count change, and for those that did, they did not find intervention effects. Alcohol use is also a dynamic behaviour, and change in the pattern of use due to intervention may not have been present long enough to lead to enduring change in viral load and/or CD4 count. Further, CD4 count measured at baseline may not be a good predictor of change and CD4 nadir maybe a better predictor of future CD4 change [59, 60]. The adherence measures in the studies reviewed were self-report or pill count, but these have also been shown to be unreliable, with antiretroviral drug levels being a better and more reliable assessment of adherence.

HIV infection is associated with other social challenges such as poverty, unemployment and isolation, and all have been shown to independently influence treatment outcomes [61]. Apart from these social factors, mental disorders such as depression, anxiety and posttraumatic stress disorder (PTSD) are common comorbidities and can influence treatment response [62]. Depression, anxiety and PTSD are associated with alcohol use, with research findings suggesting a shared neurobiological basis prompting recommendations for transdiagnostic interventions that target alcohol use, adherence and these mental disorders [63]. Further, psychosocial interventions for alcohol and depression may also work for dually diagnosed patients [64]. Complications of alcohol use include liver damage, hepatocellular carcinoma and hepatitis C, and these can all affect an individual’s ability to metabolise antiretroviral drugs and can increase the propensity to adverse effects [65–67].

A number of limitations deserve mention. Most important is the lack of standardised measures of alcohol use outcomes, whether frequency or quantity. Biochemical measures of alcohol use, such as the gamma glutaryl transferase, phosphotidyl ethanol or mean corpuscular volume, are recommended, in addition to self-report instruments. Given that the effects of alcohol on PLWH are not only related to effects on adherence, risky sexual behaviours and virological control, but are also associated with immunosuppressant and deleterious effects on the liver, these outcomes need to be included as treatment targets. Further, the studies in this review included participants with different levels of alcohol use which may affect the effects of an intervention. More severe users, including those with dependency, may require different doses of an intervention and perhaps adjunctive pharmacological therapies.

Our search was recent and comprehensive and encompassed electronic searches of key databases and a search of reference lists of included studies and relevant reviews for additional studies. It is unlikely that any studies were missed. To reduce the potential for bias, two review authors independently undertook the selection of studies, extraction of data and assessment for the risk of bias. We could have obtained more data if we had contacted authors and requested additional data, but it was not possible owing to time constraints. Finally, using GRADEpro to assess the quality of evidence would have given more robust results; however, the heterogeneity in outcomes made it difficult to tease out outcomes for inclusion using GRADEpro [65].

To our knowledge, this is the fourth systematic review to assess the evidence for psychological interventions for AUDs in PLWH. Brown et al. [21] raised similar concerns pertaining to the selection of outcome measures and the variation in study methodologies in their review. A review by Samet et al. [64] found limited evidence for the effectiveness of behavioural interventions, a finding replicated in this review. However, a recent review by Scott-Sheldon et al. [56] found behavioural interventions to be effective in reducing alcohol consumption, risky sexual behaviour and viral load in PLWH. Our review compared with that of Scott-Sheldon employed a broader search strategy, included more recent studies and rigorously assessed the risk of bias assessment [56]. We believe our findings are consistent with previous reviews in finding little evidence for effectiveness of psychological interventions for AUDs in PLWH.

## Conclusion

This systematic review did not reveal large or sustained intervention effects of psychological interventions for either primary alcohol use or secondary HIV-related outcomes. Owing to a high degree of methodological heterogeneity, a meta-analysis was not performed. Our review did not reveal significant intervention effects for both primary and secondary outcomes. There is, therefore, a need for effectiveness studies of psychological interventions for AUDs in PLWH in samples that include analyses that are disaggregated by the level of alcohol consumption, gender and age. Further, there is need to standardise alcohol use outcome measures across studies and include biomarkers, as the majority of studies have used self-report assessments that are prone to social desirability bias. Studies should also take the presence of comorbidities, such as depression, into account as they are likely to impact on intervention outcomes.

## Data Availability

Additional materials to this manuscript are available.

## Abbreviations

ART: Antiretroviral treatment
ARV: Antiretroviral
AUDIT: Alcohol Use Disorders Identification Test
AUDIT-C: Alcohol Use Disorders Identification Test-Consumption
AUDs: Alcohol use disorders
CBT: Cognitive behavioural therapy
HIV: Human immunodeficiency virus
MI: Motivational interviewing
PLWH: People living with HIV/AIDS
STI: Sexually transmitted infections
TAU: Treatment as usual
